# *Vitreoscilla* Hemoglobin Improves Sophorolipid Production in *Starmerella Bombicola* O-13–1 Under Oxygen Limited Conditions

**DOI:** 10.3389/fbioe.2021.773104

**Published:** 2021-10-26

**Authors:** Jun-feng Li, Hong-fang Li, Shu-min Yao, Meng-juan Zhao, Wen-xun Dong, Sheng-kang Liang, Xing-yong Xu

**Affiliations:** ^1^ Shandong Provincial Key Laboratory of Biochemical Engineering, Qingdao Nucleic Acid Rapid Detection Engineering Research Center, College of Marine Science and Biological Engineering, Qingdao University of Science and Technology, Qingdao, China; ^2^ College of Life Science, Qufu Normal University, Qufu, China; ^3^ Key Laboratory of Marine Chemistry Theory and Technology, Ministry of Education, Ocean University of China, Qingdao, China; ^4^ Fourth Institute of Oceanography, Ministry of Natural Resources, Beihai, China

**Keywords:** sophorolipids, *Vitreoscilla* hemoglobin, fermentation, oxygen utilization efficiency, *Starmerella bombicola*

## Abstract

Sophorolipids (SLs) are homologous microbial secondary metabolites produced by *Starmerella bombicola* and have been widely applied in many industrial fields. The biosynthesis of SLs is a highly aerobic process and is often limited by low dissolved oxygen (DO) levels. In this study, the *Vitreoscilla* hemoglobin (VHb) gene was transformed into *S. bombicola* O-13–1 by homologous recombination to alleviate oxygen limitation. VHb expression improved the intracellular oxygen utilization efficiency under either oxygen-rich or oxygen-limited conditions. In shake flask culture, the production of SLs was higher in the recombinant (VHb^+^) strain than in the wild-type (VHb^−^) strain, while the oxygen uptake rate of the recombinant (VHb^+^) strain was significantly lower than that of the wild-type (VHb^−^) strain. In a 5 L bioreactor, the production of SLs did not increase significantly, but the DO level in the fermentation broth of the VHb^+^ strain was 21.8% higher than that of VHb^−^ strain under oxygen-rich conditions. Compared to wide-type strains (VHb^−^), VHb expression enhanced SLs production by 25.1% in the recombinants (VHb^+^) under oxygen-limited conditions. In addition, VHb expression raised the transcription levels of key genes involved in the electron transfer chain (*NDH, SDH, COX*), TCA cycle (*CS*, *ICD*, *KDG1*) and SL synthesis (*CYP52M1* and *UGTA1*) in the recombinant (VHb^+^) strains. VHb expression in *S. bombicola* could enhance SLs biosynthesis and intracellular oxygen utilization efficiency by increasing ATP production and cellular respiration. Our findings highlight the potential use of VHb to improve the oxygen utilization efficiency of *S. bombicola* in the industrial-scale production of SLs using industrial and agricultural by-products like molasses and waste oil as fermentation feedstock.

## Introduction

Sophorolipid (SL) is a kind of glycolipid biosurfactant produced by the yeast *Starmerella bombicola* (Van Bogaert et al., 2011)*.* Typically, SLs is composed of a hydrophilic sophorose and hydrophobic saturated or unsaturated long-chain ω- or ω-1 hydroxy fatty acids (Asmer et al., 1988; Ma et al., 2020). As one of the most important glycolipid biosurfactants, SLs have been widely used in cosmetics, petroleum exploitation, environmental remediation, pharmaceuticals and other industries due to its high productivity, high surface activities, low toxicity and good environmental compatibility (Van Bogaert et al., 2007; Oliveira et al., 2014).

The biosynthesis of SLs requires both hydrophilic and lipophilic carbon sources. The former is usually glucose, and the latter is vegetable or fat oil (Shah et al., 2010). The key step in the synthesis of SLs is hydroxylation of fatty acids, which is catalyzed by cytochrome P450 monooxygenase and consumes a large amount of oxygen (Van Bogaert et al., 2009; Li et al., 2016c). In fact, the low utilization level of oxygen in the fermentation process leads to the low utilization of oil-soluble substrates and a long fermentation cycle, which further increases the cost of SLs production (Asmer et al., 1988; Yang et al., 2012). Therefore, the dissolved oxygen (DO) level is often a limiting factor for yeast cell growth and SL synthesis during the high-density fermentation (Yang et al., 2012). A higher oxygen utilization rate is beneficial for sophorolipid production by *S. bombicola* (Guilmanov et al., 2002).

*Vitreoscilla* hemoglobin (VHb), an oxygen-binding protein, can facilitate the intracellular oxygen transport and improve the utilization efficiency of oxygen, which help alleviate the limitation of low DO on fermentation (Zhang et al., 2007; Stark et al., 2012; Zhang et al., 2014; Stark et al., 2015). The heterologous expression of VHb gene (*vgb*) in various hosts can improve cell growth, bioremediation, protein synthesis and metabolite synthesis (Setyawati et al., 2007; Wang et al., 2012; Stark et al., 2015). The expression of VHb raises cell density of *vgb*-bearing *Gordonia amarae* and improves the production of trehalose lipid biosurfactants (Dogan et al., 2006). The production of rhamnolipid biosurfactant is enhanced by 10-fold in the *vgb*-recombinant strain (PaJC) of *Pseudomonas aeruginosa* compared to its wild-type strain (Kahraman and Erenler, 2012). The heterologous expression of VHb is also particularly beneficial to polysaccharide-producing microorganisms grown in highly viscous broth (Li et al., 2016b; Liu et al., 2017). The bio-synthesis of SLs is an energy-requiring process that consumes a large amount of oxygen (Van Bogaert et al., 2009), so it is necessary to construct a recombinant VHb-expressing strain of *S. bombicola* to improve SLs production using fat and oil feedstock.

In the previous research, we had utilized the cheap and easily available raw materials like cane molasses and waste fried oil instead of expensive carbon sources (glucose and vegetable oil) as fermentation feedstock for biosynthesis of SLs in order to reduce the cost of SLs production (Li et al., 2018)*.* In this study, the *vgb* gene encoding VHb was introduced into a sophorolipid-producing strain of *S. bombicola* O-13–1, and yeast cells were grown under either oxygen-deficient or oxygen-rich condition. The effects of VHb expression on several key parameters, such as biomass, glucose consumption, dissolved oxygen and sophorolipid production were investigated. The mechanism of VHb in sophorolipids synthesis was also addressed. This study will provide valuable evidence for resolving the problems caused by low levels of dissolved oxygen in submerged fermentation to improve the production of sophorolipid by *S. bombicola*.

## Materials and Methods

### Strains, Plasmids and Media

The wild-type strain of *Starmerella bombicola* (O-13–1) was used as the parent strain (VHb^−^), which was isolated from petroleum-contaminated soil in Shengli Oilfield, Dongying, Shangdong Province, China (Li et al., 2018). *Escherichia coli* DH5α was used as a host for the amplification of recombinant plasmids and was purchased from Tiangen Biotech (Beijing) Co., Ltd., China. Plasmid pFL4a containing hygromycin B resistance gene *hpt*II was kindly provided by Dr. Zhe Chi (Ocean University of China, Qingdao, Shangdong province, China). The plasmid pMD19-T (Simple) vector was purchased from TaKaRa Biotechnology (Dalian, China). *E. coli* transformants were selected in Luria-Bertani (LB) medium containing 100 μg/ml of ampicillin. Yeast transformants were grown in the YPD medium containing 500 μg/ml of hygromycin B. The primers and fragments were synthesized and sequenced by Tsingke Biological Technology (Beijing, China) (Table 1). LB medium contained 1% tryptone, 0.5% yeast extract, and 1% NaCl, with a pH 7.0–7.4. Seed medium (YPD) contained 1% yeast extract, 2% peptone, and 2% dextrose. Fermentation medium (w/v) consisted of 6% glucose, 6% vegetable oil, 1% yeast extract, 2% peptone, 0.5% sodium citrate, 0.4% MgSO_4_·7H_2_O, 0.2% (NH4)_2_SO_4_, 0.2% KH_2_PO_4_, 0.01% NaCl, and 0.01% CaCl_2_. Glucose and soybean oil were sterilized separately and added to the medium before fermentation.

**TABLE 1:** Primers used for plasmid construction and verification.

Genes	Primers (5′→3′)^a^	Restriction sites	Overlap
18s rDNA1-F	GAA​TTCACT​CCT​TGG​TCC​GTG​TTT​CAA​GAC​GGG	EcoR I	
18s rDNA1-R	*GTG​CCGAAT​ATTAGGTC​GAC​GGA​TCC* AGA​TTG​TAA​CGG​CGA​GTG​AAC​AGG​C	Ssp I, Sal I, BamH I	i
18s rDNA2-F	*CCG​TTA​CAA​TCTGGA​TCC​GTC​GAC* CTAAT​ATTCGG​CAC​CTT​AAC​TCC​GCG​TTC​GGT​T	BamH I, Sal I, Ssp I	i
18s rDNA2-R	GCA​TGCTTG​CCT​GCG​CGA​GTA​TTT​GGG​TGG​AAA​ACC​CAT​A	Sph I	
*HPT*-F	GGA​TCCGGT​GCT​TAG​GGT​GCG​TGT​GCA​AGG	BamH I	
*HPT*-R	*GCG​GTG​AGT​TCA​GGC​TTT​TTC​AT*TTT​TTC​TGG​TTT​GGA​GGA​CCT​TG		ii
*PGK*p-F	*CCA​AGG​TCC​TCC​AAA​CCA​GAA​AAA​A* **ATG**AAA​AAG​CCT​GAA​CTC​ACC		ii
*PGK*p-R	GTC​GACGA​GGG​CAA​AGA​AAT​AGA​GTA​GAT​GCC​GAC​CGG​GAT​C	Sal I	
*GAPD*p-F	AAT​ATTTCA​GGT​GCC​ACA​CGC​GCA​TTA​ATC​G	Ssp I	
*GAPD*p-R	GTC​GAATG​CAT​ACG​CGT​CAA​TTG​ATT​TCT​CCT​AAT​AGG​CTG​TCA​GC	Sal I	
*VGB*-F	TTC​GAA **ATG**CTC​GAC​CAG​CAG​ACC​A	BstB I	
*VGB*-R	TGC​GCA **AAT**GAG​TTG​ACG​GAC​TCG​C	Mlu I	

a The restriction sites are underlined; the start and stop codon are shown in bold; an overlapping sequence of two primers are marked with italic and the same Roman.

### Vector Construction

**FIGURE 1:**
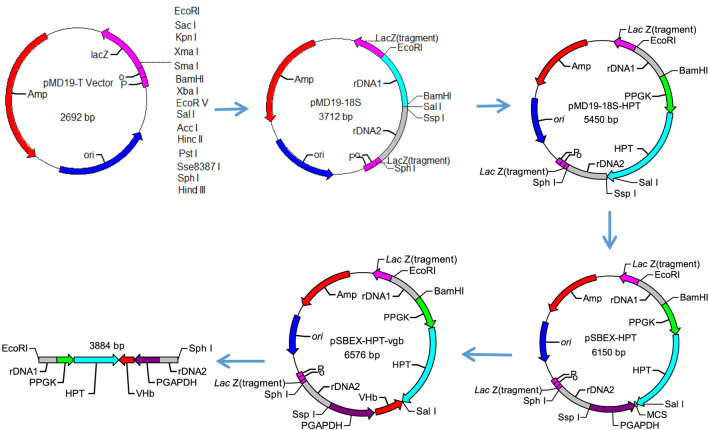
Construction of the plasmid pSBEX-HPT-vgb used for *S. bombicola* transformation.

In order to express *Vitreoscilla* hemoglobin in *S. bombicola* O-13–1, the plasmid pSBEX-HPT-Vgb was constructed based on the vector pMD-19 (Figure 1), which contained 441 bp *vgb* fused downstream of the strong constitutive glyceraldehyde-3-phosphate dehydrogenase gene (GAPDH) promoter from strain O-13–1. Briefly, genomic DNA of *S. bombicola* was used as a template for PCR amplification using two pairs of primers 18S rDNA 1-F/18S rDNA 1-R and 18S rDNA 2-F/18S rDNA 2-R that were designed as homologous recombination sites according to the DNA sequence of *S. bombicola* (GenBank accession BCGO00000000). The PCR amplification products were purified and ligated to the pMD19-T vector, and the resulting construct was named pMD19-18S. The HPT expression cassette consisted of a strong constitutive *Starmerella* phosphoglycerate kinase (PGK) gene promoter (which was amplified with the primer *PGK*p-F/*PGK*p-R), the hygromycin B phosphotransferase gene (HPT) and poly A (which was amplified with the primer *HPT*-F/*HPT*-R from the vector pFl4a) (Chi et al., 2019); and it was inserted into pMD19T-18S to yield the plasmid pMD19T-HPT-18S. In addition, the exogenous gene expression cassette consisted of the strong constitutive *Starmerella* glyceraldehyde-3-phosphate dehydrogenase (GAPDH) gene promoter (which was amplified with the primer *GAPD*p-F/*GAPD*p-R), multiple clone sites (MCS) and poly A; and it was inserted into pMD19T-HPT-18S to yield the expression vector pSBEX-HPT. After codon optimization, the nucleotide sequence of VHb gene (*VGB*, 441 bp fragment) was synthesized by Synbio Technologies (Jiangsu, China) and then amplified using PCR with the primer *VGB*-F/*VGB*-R carrying *BstB* I and *Mlu* I sites on each end. The PCR products were purified and ligated into the linearized vector pSBEX-HPT to form plasmid pSBEX-HPT-vgb, which was then transformed into *E. coli* DH5α to obtain the vgb-bearing *E. coli*, named DH5α-SBEX-HPT-vgb.

### Transformation of Yeast by Electroporation

The transformation of yeast was carried out by electroporation (Manivasakam and Schiest, 1993). The recombinant plasmids pSBEX-HPT-vgb were linearized with restriction enzymes *Sph* I and *EcoR* I (Figure 1). The linearized recombinant expression vectors were purified by agarose gel electrophoresis and transformed into wild-type *S. bombicola* strain (VHb^−^) using the homologous transformation system. The preparation of competent cells of *S. bombicola*, electroporation of linearized recombinant expression fragments and screen of transformants were carried out according to the methods described elsewhere (Chi et al., 2012). The DNA of the transformants were extracted, and the successful transformation of the *vgb* gene was verified using PCR with *VGB*-F and *VGB*-R primers. Putative transformants were grown on YPD plates without hygromycin B for five rounds. After growth on nonselective YPD plates, those tested transformants can stably maintain hygromycin B resistance.

### Identification of *Vitreoscilla* Hemoglobin (CO-Difference Spectrum Analysis)

The presence of the vgb gene was confirmed by PCR analysis (Ye et al., 2016). The activity of the expressed VHb protein was determined by CO-difference spectral analysis (Li et al., 2016b). Briefly, VHb^+^ and VHb^−^ yeast cells were harvested from the fermentation medium by centrifugation at 5,000 rpm for 15 min. 5 g of the cells were washed twice with 0.85% saline solution. Around 1.5 g of the cells were resuspended with 30 ml phosphate buffer (pH 7.0) after centrifugation and then disrupted with an ultrasonifier (Liao et al., 2014). After centrifugation at 8,000 rpm for 5 min at 4°C to remove cell debris, the supernatant was reduced with excess sodium dithionite (2.5 mg/ml) and divided into two aliquots. One aliquot was bubbled with CO for 2 min and the other with air for the same period of time. The samples were then scanned in the range 400–460 nm on a spectrophotometer. The CO-difference spectrum for each sample was calculated by subtracting the absorbance of the air-bubbled sample from that of the CO-bubbled sample (Su et al., 2010)

### Determination of Oxygen Uptake Rate

The oxygen uptake rate (OUR) was calculated according to the equation (Garcia-Ochoa et al., 2010): , where C_L_ is dissolved oxygen (DO) in the broth, t is cultured time. The concentrations of DO in the medium were determined using a dissolved oxygen sensor (InPro6860i/12/120/mA/HD, Mettler-Toledo) and was expressed as percent saturation (Ozbek and Gayik, 2001). The DO concentrations were recorded every 10 s, and the experiments were repeated two times.

### Bioreactor-Scale Fermentation

To study the effect of VHb expression on *S. bombicola* O-13–1 under both the oxygen-enriched and oxygen-limited conditions, we compared the oxygen uptake rate, biomass, glucose consumption and sophorolipid production between the transformant VHb^+^ and the wild type VHb^−^ in a 5 L bioreactor (BIOTECH-5BG, Shanghai Baoxing Bio-engineering, China) containing 2 L of fermentation broth. The fermentation broth was cultured under oxygen-enriched conditions (rotation speed 400 rpm, aeration 1.0 vvm) and oxygen-limited conditions (rotation speed 350 rpm, aeration 1.0 vvm). The sampling interval was 4 h.

### Analytical Methods

Biomass was determined by measuring cell dry weight after removal of SLs and other hydrophobic substrates in the fermentation broth (Felse et al., 2007). SLs were extracted from the fermentation broth according to the method of Hu and Ju (2001). Briefly, 10 ml of fermentation broth was extracted two times with an equal volume of ethyl acetate as solvent. The solvent was then centrifuged at 8,000 rpm for 10 min at 4°C. The organic phase was vacuum-dried at 40°C to remove ethyl acetate. The residues were washed with 10 ml hexane to remove the remaining oil. The crude SLs were obtained after vaporizing the residual hexane at 40°C under vacuum. Glucose content was measured using the 3,5-dinitrosalicylic acid (DNS) method (Wang et al., 2010).

### Expression of Several Selected Genes Using Quantitative Real-Time PCR

Total RNA was isolated using Fungal RNA Kit (OMEGA bio-tek, Norcross, GA, United States). The cDNA was synthesized by the Thermo Scientific Revert Aid First Strand cDNA Synthesis Kit. The gene primers are listed in Table 2. β-tubulin gene was used as an internal control. The PCR conditions were described as follows: 95 C for 10 min, 30 cycles of 95 C for 3 s, 56 C for 30 s, and 72 C for 60 s. The melting curve was analyzed to evaluate the specificity of primers used for RT-qPCR. All amplifications were performed in triplicates.

**TABLE 2:** Primers and relevant information of reference and target genes for quantitative real-time PCR.

Genes	Name	Primers (5′-3′)
*NDH*	NADH dehydrogenase	F: AAC​TCA​ATC​CCT​CGT​CGT​CAG
		R: AAT​AGC​CTG​TCC​ACT​CTT​TCC​C
*SDH*	Succinate dehydrogenase	F: GCG​TGA​GTT​TTC​AAC​GGT​GG
		R: ACC​GAC​GGG​AGG​GGT​TAC​TAT
*COX*	Cytochrome c oxidase	F: GGC​ATT​TGG​TCG​GGT​TCA​TA
		R: GCC​CAT​CTT​GAC​TCG​CTA​CTG​T
*KDG1*	Alpha-ketoglutarate dehydrogenase	F: TGG​ATT​TCC​GCC​AAT​ACC​G
		R: GCT​GAA​AAC​ACC​AAA​CAC​GAG
*ICD*	Isocitrate dehydrogenase	F: TTC​ATG​CGG​AGG​TTA​CGA​CA
		R: GCC​TCG​CCA​ATG​TAA​AGA​CG
*CS*	Citrate synthase	F: GTC​TAC​TCA​CCA​ACT​CAA​TCC​CTC
		R: CCA​ATA​GCC​TGT​CCA​CTC​TTT​C
*CYP52M1*	Cytochrome P450 monooxygenase	F: GGG​TCC​GTT​TGA​AAG​CGT​AAT
		R: TTG​TAG​CCC​GTG​ATG​GTT​CG
*UGTA*	UDP-glucosyltransferase	F: TGG​TTC​ATA​GCG​AGT​TTC​TTT​GC
		R: CTG​GCT​GGA​TTT​GTT​TAG​GGG
*VGB*	*Vireoscilla* hemoglobin	F: CGA​GAA​CTG​TCA​TAG​CAA​GAG​CC
		R: ACA​TCA​TCA​AGG​CTA​CCG​TTC​C
Tubulin	Tubulin Z cytoskeleton	F: TGA​TGA​GAC​GGG​CTG​GGA​AT
		R: ACC​GTT​ACT​GAA​CCT​TAC​AAT​GCC

Relative expression levels of the selected genes were calculated using the 2^−∆∆Ct^ method (Zhang et al., 2011), in which C_T_ is threshold period, and Tubulin was used as the reference gene. In this study, the genes in wild-type strains VHb^−^ were used as control to normalize expression levels. Therefore, the comparative expression level of each gene in the strains VHb^−^ was set to be 1.

### Statistical Analysis

Statistical analysis was performed with Student’s *t*-test. Differences were considered statistically significant when *p*-values were <0.05 in a two-tailed analysis.

## Results and Discussion

### Confirmation of the *vgb*-Bearing *S. bombicola*

**FIGURE 2:**
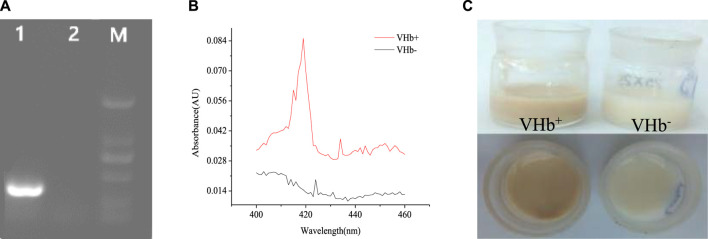
Phenotypic analysis of the *S. bombicola vgb* strain. **(A)** Electrophoresis analysis of genomic DNA extracted from wild-type strains *Vitreoscilla* hemoglobin (VHb^−^) and transformants (VHb^+^) of *S. bombicola*. Lane 1: VHb^+^ (441 bp); Lane 2: VHb^−^(wild-type strain); Lane M: DL 2000 DNA marker (2000 bp, 1,000 bp, 750 bp, 500 bp, 250 bp, 100 bp); **(B)** CO-difference spectra of the *vgb*-bearing *S. bombicola* strain (VHb^+^) and the wild-type strain (VHb^−^); **(C)** The color of the VHb^+^ and VHb^−^ strains.

To confirm the integration of the *vgb* gene into the genomes of *S. bombicola* O-13–1, genomic DNA of transformants was independently isolated and detected by PCR with the special primer pair listed in Table 1. Agarose gel electrophoresis of PCR products showed a clear band of the *vgb* fragment (441 bp) in the VHb^+^ transformant, but not in the VHb^−^ strain (Figure 2A), indicating that exogenous *vgb* gene was successfully transformed into the host strain. The VHb^+^ has a maximum absorption at 419 nm when CO is bound, so the biological activity of the VHb was verified by CO-difference spectra (Geckil et al., 2001). The characteristic absorption peak at 419 nm was found in the CO-spectra of crude extracts of the *vgb*-bearing *S. bombicola*, but not in the wide type strain (Figure 2B), suggesting that biologically active VHb was successfully expressed in the VHb^+^ strain. Similarly, the expression of VHb was also identified in *Pichia pastoris* (Wu and Fu, 2011) and *Aureobasidium melanogenum* P16 (Xue et al., 2019) by CO-difference spectral analysis. In addition, the VHb^+^ cells appeared light brown (the color of the VHb), while the VHb^−^ cells remained milk white without change (Figure 2C). These results demonstrated that *vgb* was successfully expressed in *S. bombicola* O-13–1 transformants (VHb^+^) and exhibited its biological activity.

### Effects of *Vitreoscilla* hemoglobin Expression on Sophorolipids Fermentation of *S. bombicola* Cultivated in Shake Flasks

**FIGURE 3:**
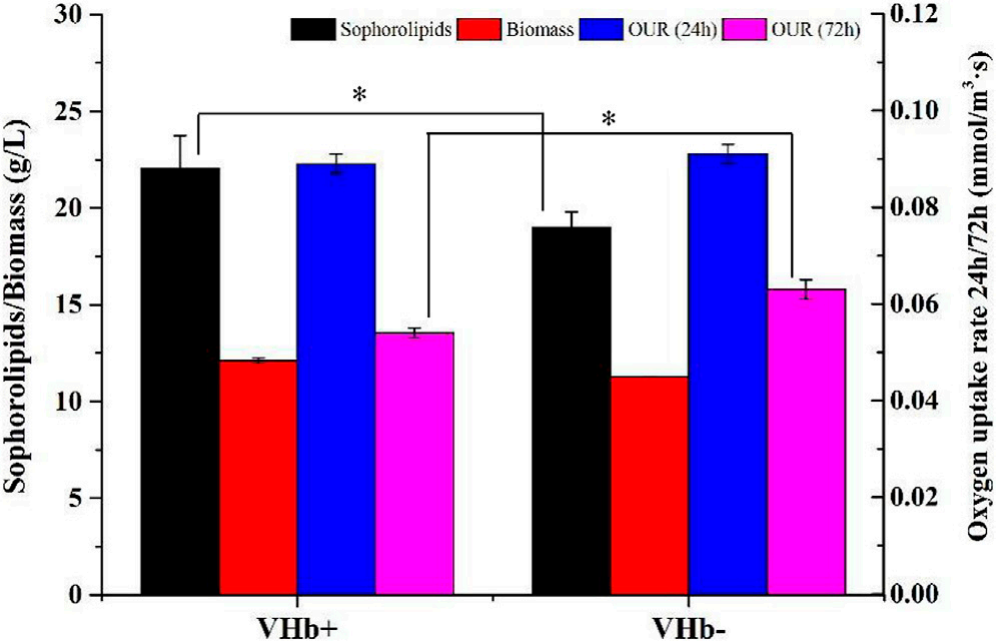
Comparison of sophorolipid fermentation between the VHb^+^ and VHb^−^ strains cultivated in shake flasks (*p < 0.05).

The sophorolipid production, biomass and oxygen uptake rate (OUR) of the recombinants and wild-type strains at 24 and 72 h were showed in Figure 3. At 24 h, there was no difference in the biomass and OUR between the recombinant strains (VHb^+^) and wild-type strains (VHb^−^). At 72 h, the sophorolipids production of the recombinant strains (VHb^+^) was higher than that of the wild-type strain (VHb^−^), and the OUR of the VHb^+^ was lower than that of the VHb^−^. The results indicated that the expression of VHb could promote the utilization efficiency of intracellular oxygen, thereby reducing the OUR of the recombinant strains (VHb^+^). The result is consistent with other studies (Huang et al., 2014; Wang Q. et al., 2018).

### Effects of *Vitreoscilla* hemoglobin Expression on Sophorolipidss Production of *S. bombicola* Cultivated in a 5 L Bioreactor

**FIGURE 4:**
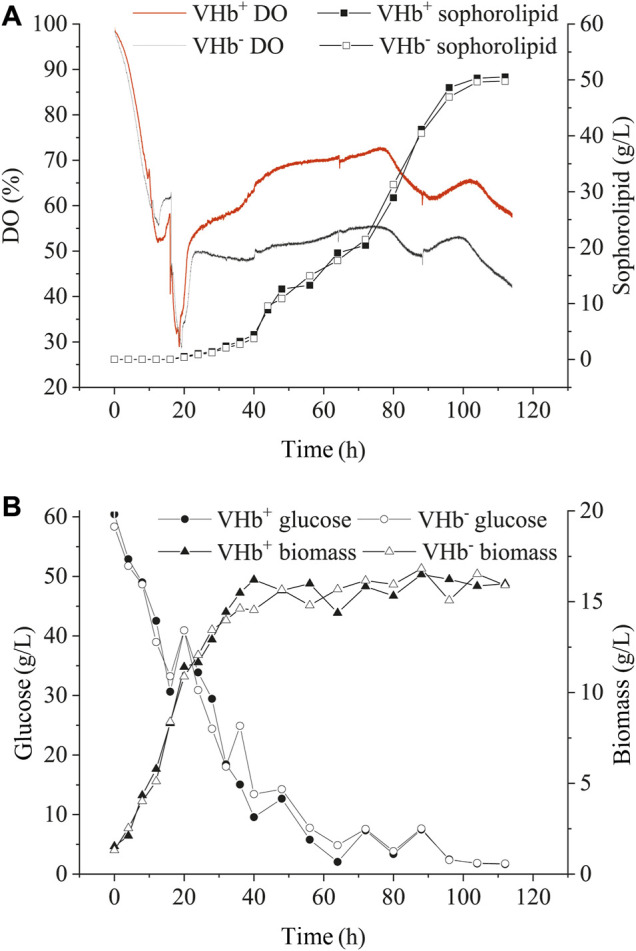
Effects of VHb expression on DO content and SLs production **(A)**, residual sugar and biomass **(B)** under oxygen-rich conditions.

The transformants (VHb^+^) and wild-type strains (VHb^−^) were incubated in a 5 L bioreactor under either oxygen-limited or oxygen-rich conditions. Under the oxygen-enrich condition, the recombinant strains (VHb^+^) had similar biomass, sophorolipid production and glucose consumption with the wild-type strains (VHb^−^) during the entire fermentation stage (Figure 4B). Interestingly, though there was no significant change in DO content of the fermentation broth between VHb^+^ and VHb^−^ strains at the early stage of fermentation (within 20 h, Figure 4A), the DO content of the fermentation broth for the VHb^+^ strains raised to be 21.8% higher than that for VHb^−^ strains at the late stage (40–112 h) of fermentation (Figure 4A). Previous researches have shown that VHb expression had no significant effects on the growth and metabolism of Ganoderma lucidum and *Pseudomonas aeruginosa* under oxygen-rich conditions (Li et al., 2016a, Li et al., 2016b; Geckil et al., 2001). However, other studies have also shown that VHb expression enhanced the biomass and yield of VHb recombinant strain under same conditions (Dogan et al., 2006; Gao et al., 2018).

**FIGURE 5:**
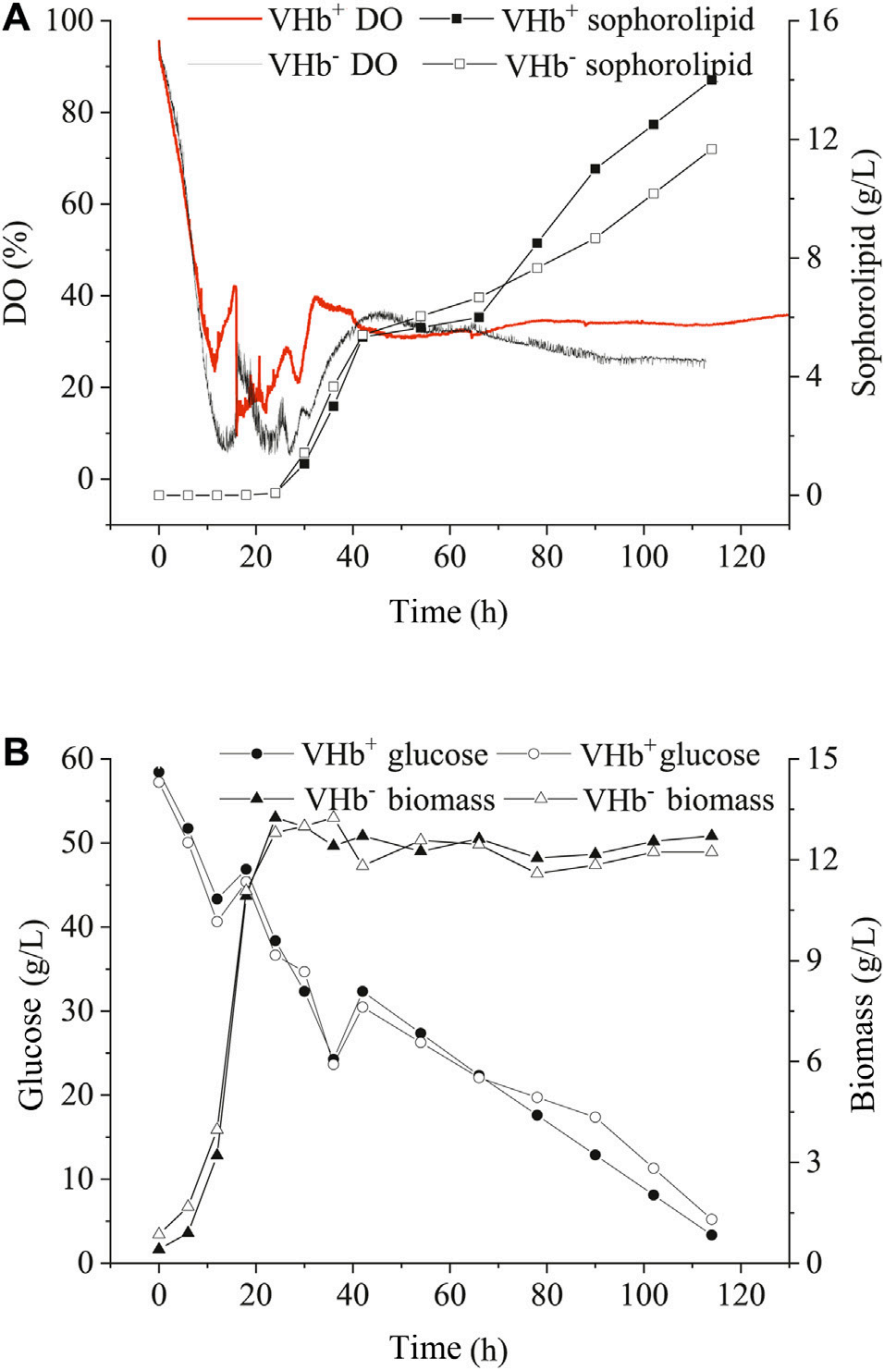
Effects of VHb expression on DO content and sophorolipid production **(A)**, the residual sugar and biomass **(B)** under oxygen-limited conditions.

Under oxygen-limited conditions, the changes in SLs production, glucose consumption and DO concentration of the fermentation broth were similar between the recombinants and wild-type strains during the early stage (within 40 h) of fermentation (Figure 5). After 72 h, SLs production, glucose consumption and biomass in the recombinants significantly increased compared with those in the wild-type strains, however, the consumption of DO in the recombinants decreased compared with that in the wild-type strains. In the end of fermentation, the production of SLs in the recombinants (VHb^+^) boosted by 25.1% compared with that of the wild-type strains (VHb^−^). Moreover, oxygen consumption in the recombinant strains (VHb^+^) was obviously lower than that in the wild-type strains (VHb^−^), especially in the late stages of fermentation (Figure 5A).

In the early stage of fermentation, the recombinant strains (VHb^+^) and wild-type strains (VHb^−^) showed no significant difference in OUR under the oxygen-rich or oxygen-limited conditions. However, in the later stage of fermentation, the DO content in the fermentation broth of the VHb^+^ strains was higher than that of the VHb^−^ strains under oxygen-rich or oxygen-limited conditions, indicating that the expression of VHb could decrease oxygen consumption and promote the yield of SLs by the recombinants (VHb^+^) (Figures 4, 5). The results are consistent with previous reports concerning fungi and bacteria. For example, the concentration of pullulan and productivity were greatly enhanced by overexpression of VHb in *A. melanogenum* P16 under oxygen-limited conditions (Xue et al., 2019). The biomass and protein production increased in VHb-expressing Aspergillus sojae (Mora-Lugo et al., 2015) and Schwanniomyces occidentalis (Suthar and Chattoo, 2006). The bacterial cellulose production enhanced in *vgb-*bearing *Gluconacetobacter xylinus* under oxygen-limited conditions (Liu et al., 2018), and surfactin production improved by 24 and 51% in the VHb-expressing *Bacillus subtilis* THY-15/Pg3-srfA cultivated in the flasks and the fermentor, respectively (Wang Q. et al., 2018). These results indicated that the function of VHb might vary in different microorganisms under different growth conditions.

SLs production requires a certain amount of oxygen either in submerged fermentation (Guilmanov et al., 2002; Zhang et al., 2018) or solid-state fermentation (Jiménez-Peñalver et al., 2016; Jiménez-Peñalver et al., 2018). In this study, for the recombinants (VHb^+^) cultivated in shake flasks, VHb expression did not raise the OUR during the first 24 h but reduced the absorption of oxygen by the recombinants (VHb^+^) (Figure 3); in addition, the production of SLs was higher in the recombinant strains (VHb^+^) than in the wild-type strains (VHb^−^). The similar results were found in the strain cultivated in the 5 L fermentor (Figures 4, 5). The reason that the OUR of the recombinant was lower and the intracellular oxygen utilization efficiency was higher than that of the reference strain on aerobic metabolism was not completely understood. Some researchers think that expression of VHb can improve the level of intracellular dissolved oxygen by enhancing oxygen delivery, thus improving the respiration and energy metabolism of the cell (Wei and Chen, 2008; Frey et al., 2011). The results above demonstrated that the VHb gene was successfully transformed in the yeast strain *S. bombicola* O-13–1, and VHb expression improved the oxygen utilization efficiency. Meanwhile, the expression of *vgb* in *S. bombicola* O-13–1 provided a new strategy to promote SLs production in highly viscous fermentation systems.

### Effects of *Vitreoscilla* hemoglobin Expression on the Expression of Several Key Host Genes

**FIGURE 6:**
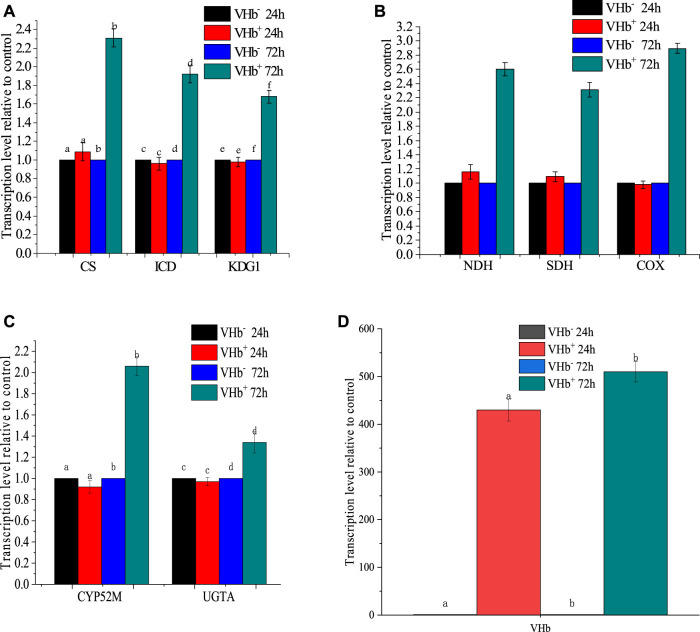
Transcription levels of key genes involved in the TCA cycle, respiratory chain and sophorolipid biosynthesis at 24 and 72 h of fermentation. The transcription levels of genes in the recombinant strains were normalized to the transcription level of genes in the wild-type strains. **(A)** transcription levels of *CS*, *ICD*, and *KDG1*; **(B)** transcription levels of *NDH*, *SDH*, and *COX*; **(C)** transcription levels of *CYP52M* and *UGTA*; and **(D)** expression level of VHb.

The presence of VHb can regulate gene expression in its host (Stark et al., 2015). In this study, expression levels of several host genes, including two genes (CYP52*M1* and *UGTA1*) involved in SLs biosynthesis, three genes (*CS*, *ICD*, and *KDG1*) involved in tricarboxylic acid (TCA) cycle, and three genes (*NDH, SDH, COX*) involved in electron transport chain (ETC) and ATP production, were determined in the transformant strains and wild-type strains using qRT-PCR at 24 and 72 h (Figure 6). At 24 h, all genes above were expressed approximately at the same levels in the transformants and wild-type strains. At 72 h, however, the expression levels of these genes were higher in the transformants than in the wild-type strains. The results might partially explain the phenomenon that VHb expression did not improve the OUR of the yeast in the first 24 h but reduced oxygen uptake during SLs production. Similarly, VHb expression enhanced natamycin production in recombinant strains of *Streptomyces gilvosporeus* compared to wild-type strains (Wang H. et al., 2018), and ployhydroxybutyrate (PHB) production was much higher in *vgb*-bearing strains Reh01 than in wild-type strains of *Cupriavidus necator* H16 (Tang et al., 2020). These results indicated that expression of *vgb* gene greatly raised the production of surfactants in the later stage of fermentation.

Cytochrome P450 monooxygenase is a key enzyme in SLs synthesis that hydroxylates the end of fatty acids and controls the chain length of the SLs hydroxy fatty acid tail (Van Bogaert et al., 2009). Uracil diphosphate (UDP)-glucosyltransferase is an enzyme responsible for the first glucosylation step in the SLs biosynthetic pathway (Saerens et al., 2011). In this study, the expression of two genes, including *cyp52m1* encoding cytochrome P450 monooxygenase and *ugta1* encoding uracil diphosphate glucosyltransferase, were determined to evaluate the effects of VHb expression on the biosynthesis of SLs (Figure 6A). VHb expression increased the transcriptional levels of *cyp52m1* and *ugta1* by 2.03 folds and 1.31 folds, respectively, compared to the wild-type strains.

A sufficient supply of oxygen is crucial for cellular respiration. In an electron transport chain (ETC), electrons can be transferred from electron donors (e.g., NADH or FADH_2_) into oxygen as an electron acceptor to release protons for ATP synthesis, tricarboxylic acid (TCA) cycle is the main source of electron donors for the ETC. The biosynthesis of SLs is an energy-requiring process. The additional ATP for SLs production can be provided by enhancing the ETC and TCA cycle through heterologous expression of VHb. NADH dehydrogenase (NDH), succinate dehydrogenase (SDH) and cytochrome c oxidase (COX) are three main respiratory oxidases in *S. bombicola* (Freel et al., 2015). The transcription levels of *COX*, *NDH* and *SDH* in the recombinants promoted by 2.89, 2.55 and 2.28 folds compared to those in the wild-type strains, respectively (Figure 6B), indicating that the expression of VHb could boost cellular respiration and require more electron donors (NADH or FADH). Meanwhile, it suggested that VHb could provide oxygen directly to the terminal oxidases. This is consistent with the characteristics of VHb, which interacts with terminal respiratory oxidases to generate an efficient electron transfer for promoting energy generation (Stark et al., 2015). Given that citrate synthase (CS), isocitrate dehydrogenase (ICD), and alpha-ketoglutarate dehydrogenase (KGDH, coded by gene *KDG1*) are enzymes involved in the production of electron donors in the TCA cycle, the transcription levels of *CS*, *ICD* and *KDG1* were also measured (Figure 6C). The expression of these three genes in the recombinants improved by 2.27, 1.90 and 1.60 folds compared to those in the wild-type strains, respectively, indicating that the expression of VHb enhanced the transcription of genes involved in the ETC and TCA cycle.

As shown in Figure 6D, VHb had a higher expression level at 24 h of fermentation because the promoter used for expression was constitutive. However, there is no significant difference in the expression levels of these key genes involved in ETC, TCA cycle and SLs synthesis compared with the wild-type strain (VHb^−^). At 72 h, though the expression level of VHb only raised by 1.18 folds, its expression significantly increased the expression of the key genes involved in the ETC, TCA cycle and SLs synthesis. However, it is still unknown about the mechanism underlying the effects of VHb expression on aerobic metabolism in *S. bombicola*, and more detailed studies at molecular levels should be conducted in the future.

The presence and function of VHb in promoting respiration and ATP formation has been considered to be responsible for the improvements of cell growth, protein synthesis and metabolism (Stark et al., 2011). For instance, the transcription levels of *PGM*, *UGP* and *GLS* involved in polysaccharide biosynthesis were up-regulated by 1.51-, 1.55- and 3.83-fold, respectively, in vgb-bearing G. lucidum (Li et al., 2016b). Similarly, the transcription levels of key genes involved in the ETC, TCA cycle, and exopolysaccharides synthesis promoted in vgb-bearing strains (Liu et al., 2017; Xue et al., 2019).

## Conclusion

A heterologous protein VHb was successfully expressed in S. bomcicola O-13–1 and was biochemically active. Compared with the wild-type strains, the expression of VHb in the recombinants significantly enhanced the intracellular oxygen utilization efficiency without improving the production of SLs. VHb expression could up-regulate the expression of key genes involved in the ETC, TCA cycle and SLs biosynthesis by improving cellular respiration and ATP supply. The findings highlight the potential use of VHb to improve the industrial-scale production of SLs utilizing agro-industrial waste as feedstock when the bioreactor is limited by the oxygen supply.

## Data Availability

The original contributions presented in the study are included in the article/Supplementary Material, further inquiries can be directed to the corresponding author.
